# Are HIV and reproductive health services adapted to the needs of female sex workers? Results of a policy and situational analysis in Tete, Mozambique

**DOI:** 10.1186/s12913-016-1551-y

**Published:** 2016-07-26

**Authors:** Yves Lafort, Osvaldo Jocitala, Balthazar Candrinho, Letitia Greener, Mags Beksinska, Jenni A. Smit, Matthew Chersich, Wim Delva

**Affiliations:** 1 International Centre for Reproductive Health, Ghent University, Ghent, Belgium; 2 International Centre for Reproductive Health-Mozambique, Maputo, Mozambique; 3 Direcção Provincial de Saúde de Tete, Tete, Mozambique; 4 MatCH Research Unit (Maternal, Adolescent and Child Health Research Unit), University of the Witwatersrand, Durban, South Africa; 5 Discipline of Pharmaceutical Sciences, College of Health Sciences, University of KwaZulu-Natal, Durban, South Africa; 6 Wits Reproductive Health and HIV Institute, Faculty of Health Sciences, University of the Witwatersrand, Johannesburg, South Africa; 7 The South African DST/NRF Centre of Excellence in Epidemiological Modelling and Analysis (SACEMA), University of Stellenbosch, Stellenbosch, South Africa; 8 Center for Statistics, Hasselt University, Diepenbeek, Belgium

**Keywords:** HIV, Reproductive health, Female sex workers, Mozambique, Health services, Mixed methods, Availability

## Abstract

**Background:**

In the context of an implementation research project aiming at improving use of HIV and sexual and reproductive health (SRH) services for female sex workers (FSWs), a broad situational analysis was conducted in Tete, Mozambique, assessing if services are adapted to the needs of FSWs.

**Methods:**

Methods comprised (1) a policy analysis including a review of national guidelines and interviews with policy makers, and (2) health facility assessments at 6 public and 1 private health facilities, and 1 clinic specifically targeting FSWs, consisting of an audit checklist, interviews with 18 HIV/SRH care providers and interviews of 99 HIV/SRH care users.

**Results:**

There exist national guidelines for most HIV/SRH care services, but none provides guidance for care adapted to the needs of high-risk women such as FSWs. The Ministry of Health recently initiated the process of establishing guidelines for attendance of key populations, including FSWs, at public health facilities. Policy makers have different views on the best approach for providing services to FSWs—integrated in the general health services or through parallel services for key populations—and there exists no national strategy. The most important provider of HIV/SRH services in the study area is the government. Most basic services are widely available, with the exception of certain family planning methods, cervical cancer screening, services for victims of sexual and gender-based violence, and termination of pregnancy (TOP). The public facilities face serious limitations in term of space, staff, equipment, regular supplies and adequate provider practices. A stand-alone clinic targeting key populations offers a limited range of services to the FSW population in part of the area. Private clinics offer only a few services, at commercial prices.

**Conclusion:**

There is a need to improve the availability of quality HIV/SRH services in general and to FSWs specifically, and to develop guidelines for care adapted to the needs of FSWs. Access for FSWs can be improved by either expanding the range of services and the coverage of the targeted clinic and/or by improving access to adapted care at the public health services and ensure a minimum standard of quality.

**Electronic supplementary material:**

The online version of this article (doi:10.1186/s12913-016-1551-y) contains supplementary material, which is available to authorized users.

## Background

Although female sex workers (FSWs) are highly exposed to sexual and reproductive health (SRH) risks, such as HIV and other sexually transmitted infections (STI) [1–3], unwanted pregnancies [4–6] and sexual violence [7, 8], general health services are often not adapted to the specific context and needs of FSWs. When attending SRH services, such as for STI/HIV testing and care, family planning, cervical cancer screening and sexual and gender-based violence, FSWs often do not disclose as such by fear of stigmatisation and discrimination, providers do not actively assess their risk, and sometimes national guidelines provide insufficient guidance on how to offer care adapted to their high-risk profile [9]. In the wake of the HIV epidemic, and because of the key role FSWs have in it, several initiatives have been taken in the past decades to improve HIV/SRH services for FSWs, either in the form of parallel services adapted to the FSWs’ needs [10–13] or through interventions to make general HIV/SRH services more FSW-friendly [10].

The DIFFER project (Diagonal Interventions to Fast-Forward Expanded Reproductive Health) is an operational research project that aims at improving access to FSW-adapted HIV/SRH care by linking services targeted at FSWs with the general health services [14]. It is designed as a set of case studies, with the ‘case’ being a well-defined geographical area where sex work is common. One of these is the Tete-Moatize area in Mozambique. The adjacent cities of Tete and Moatize are intersected by a major transport route connecting Malawi to Zimbabwe and the port of Beira. There is a rapidly growing mining industry, attracting travellers, migrant labour and sex workers. Certain practices associated with sex work, such as publicly soliciting clients, are illegal in Mozambique, and FSWs are therefore a marginalised population.

The towns have a number of health care facilities, including 1 provincial hospital, 8 public health centres and 4 private clinics. In Moatize, a stand-alone drop-in clinic for most at risk populations offers basic HIV and SRH services during the evening and is therefore called the Night Clinic. It is operated by a non-governmental organisation, through an agreement with the district health department that supplies the drugs and medical supplies and the health staff who provides the services after hours against over-time compensation [13].

The DIFFER project applies a methodological framework for health systems research, starting with a detailed situation and policy analysis that informs the development of site and context-specific packages of interventions to strengthen SRH service delivery. These packages are then implemented and on completion of the intervention the feasibility, acceptability, effectiveness, cost-effectiveness and sustainability are evaluated. The baseline analysis applied a convergent parallel mixed methods design, combining multiple qualitative and quantitative research methods [15, 16]. We assessed both the offer and the demand side. The use of HIV/SRH services by FSWs and barriers to use were assessed through a cross-sectional survey and focus group discussions with FSWs, and its results are presented elsewhere. What HIV/SRH services are available, and to what extent they are adapted to the needs of FSWs, was assessed through a policy and situational analysis. The research questions to be addressed by this analysis were (1) what national policies exist in regards to HIV/SRH service provision, and to what extent these policies address the particular needs of FSWs; (2) what HIV/SRH services are locally provided and to what extent are they adapted to the needs of FSWs; and (3) what is according to policy makers and health providers the best model to improve access to HIV/SRH services for FSWs. The current article presents the findings of the baseline policy and situational analysis of the availability of HIV/SRH services in Tete-Moatize.

## Methods

### Study components and populations

The baseline analysis consisted of (1) a policy analysis and (2) health facility-level assessments.

The policy analysis comprised of a review of all national policy and strategy documents and guidelines on HIV/SRH programmes and interventions with key populations, and of interviewing key informant (KI) policy and decision makers using a semi-structured guide. KI were eligible if they had an important role in defining HIV/SRH/FSW strategies at either local (district or province) or national level, and included both government officials and representatives from agencies supporting the government. Topics addressed included current policies, strategies and guidelines on HIV/SRH and key populations, availability of HIV/SRH services in the public sector, access to and use of the public services by FSWs, and appreciation of different models to enhance access to services for FSWs.

All health facilities in Tete/Moatize with sex work hotspots in their catchment area were assessed. These were 5 public health centres, 1 private clinic and the Night Clinic. The public health centres fell into three different types: a type 1 small public health centre offering only basic primary health care (*n =* 1); type 2 larger centres that also offer secondary health care (*n =* 3); and a type 3 centre with both in-patient and outpatient care (*n =* 1). In addition, we assessed the gynaecology department of the public referral hospital. First, a standardised facility audit tool was completed that comprised of an interview of the facility manager, an inventory checklist filled out during the facility visit, and an observation check list filled out by an observer on another day of the audit. The tool assessed the scope, volume and capacity of existing SRH service delivery. Next, all providers of HIV/SRH care services (family planning, HIV testing services (HTS), HIV care, STI care and cervical cancer screening) at the 5 public health centres and the hospital were interviewed, using a structured questionnaire. Interview topics included practices of SRH care and attitudes towards and case management of FSWs. Lastly, to document practices conducted during HIV/SRH consultations, as reported by female users, a representative sample of women (18 years and older) attending HIV/SRH services at the 5 public health centres were interviewed after exiting the services. The target sample size was 100 to allow the measurement of practices with sufficient precision (*p =* .50, d = .15). The number of participants recruited per facility and per service was proportional to the number of women attended at the facility and service in the previous year. Participants were interviewed face-to-face using a structured CAPI (QDS™) questionnaire that addressed socio-demographic characteristics, the type of services received and appreciation of the services.

All study participants provided written informed consent and the study protocols were approved by the National Committee of Bioethics for Health in Mozambique and the Commission for Medical Ethics of the University Hospital Ghent.

### Data analysis

KI interviews were audio-recorded, transcribed and manually analysed for key concepts. Answers were deductively and selectively coded, using a theoretical framework, by topic addressed, type of HIV/SRH service and type of policy maker [17]. The information collected during the facility audits was transcribed in a spreadsheet and manually analysed. The provider and user data were analysed using Intercooled Stata version 11.0 (College Station, Texas, United States).

The results of the different research methods were triangulated to provide an answer to the research questions. Responses given by policy makers during the key informant interviews were cross-checked with the information from the policy documents and synthesized into a summary table presenting key findings per HIV/SRH service. The information from the facility audits was side-by-side compared to the responses given by providers and users on what and how services are provided, to reach an integrated conclusion.

## Results

### Policy analysis

The combined results of the policy document review and the KI interviews are summarised in Additional file 1: Table S1.

#### Policy document review

A total of 8 national documents were reviewed: one strategy (family planning), 4 finalised technical guidelines (family planning, cervical cancer screening, STI care and HIV care), 1 draft technical guideline (HIV services for most-at-risk populations) and 2 slide show presentations of a draft strategy (sexual and gender-based violence (SGBV) care and HIV prevention, diagnosis and treatment). No separate national guidelines or strategies were found for condom distribution, HIV testing services, or services for unwanted pregnancies. None of the Mozambican HIV/SRH clinical guidelines or strategies took into account the risk profile of clients and they were often not appropriate for the specific context of FSWs. For example, STI care guidelines did not include a risk assessment to decide on the treatment to give, nor did the family planning and cervical cancer screening guidelines offer advice for an adapted approach for women with a higher risk of STI. Only the slide show presentation of the draft HIV prevention, diagnosis and treatment strategy mentioned activities specifically targeting key populations.

#### Key informant interviews

Twelve people were interviewed, 8 at the central level, 2 at the provincial level and 2 at the district level. At the central level, three participants were from the government, one from a donor agency, one from a UN agency, and three from different international non-governmental organisations (NGOs). At the provincial and district levels, all participants were from the government. They had on average 12 years of experience in SRH and/or HIV programmes and had been on average 3.5 years in their current position. The key informants provided information on what HIV/SRH strategies and guidelines were in place, what HIV/SRH services were available and what developments were expected in the near future.

Services that were not yet widely available in the public sector included those for victims of SGBV, women with unwanted pregnancies, cervical cancer screening, female condoms and implants for family planning (FP). In all of these areas steps were however being taken towards improving the provision of these services. National guidelines for the provision of SGBV services were in development, cervical cancer screening and the provision of female condoms and implants were gradually being introduced, and lobbying for the legalisation of TOP was ongoing in the country.

When asked what the preferred model for enhancing access to HIV/SRH services for FSWs was, key informants were divided. Government officials tended to favour improving access to the general health services, claiming that it is a duty of the government to provide services for all, while representatives of donor agencies and international NGOs were more open to the establishment of parallel services operated by non-governmental actors. Nevertheless, government officials also accepted the presence of parallel services created in the context of internationally funded projects, as long as they are temporary until better access to the general health services is achieved.

### Facility assessment

#### Facility audits (Table 1)

**Table 1:** Facility audit findings

Type of facility	Type 1 health centre	Type 2 health centres	Type 3 health centre	Hospital	Night Clinic	Private clinic
SRH services offered						
Male condom distribution	+	3/3	+	-	+	+
Female condom distribution	+	3/3	+	-	-	-
Oral and injectable contraceptives, IUD	+	3/3	+	-	+	-
Implant	-	-	-	-	-	-
Female sterilisation	-	-	-	+	-	-
Syndromic STI care	+	3/3	+	-	+	-
Non-syndromic STI care	-	-	-	+	-	+
HTS	+	3/3	+	+	+	+
HIV care (including ART and PEP)	-	3/3	+	+	-	-
Cervical cancer screening	-	1/3	+	+	-	-
SGBV services	-	-	-	+	+	-
Conditions						
Sufficient space in the waiting area	-	-	-	*NA*	+	+
Electricity 24 h/24 h, without interruption in the past month	-	2/3	-	*NA*	-	+
Adequate water supply	-	1/3	-	*NA*	-	+
Clean and adequate toilet facilities	-	-		*NA*	+	+
Sufficient No of consultation rooms	-	-	+	*NA*	+	+
Not more than one provider in any consultation room	-	2/3	-	*NA*	+	+
Private conversation possible in all SRH consultation rooms	-	1/3	+	*NA*	-	-
Well ventilated and air conditioned consultation rooms	-	-	+	*NA*	+	+
Well illuminated consultation rooms	-	1/3	-	*NA*	+	+
Gynaecological examination tables in good conditions in all SRH consultation rooms	-	-	-	*NA*	+	+
Adequate illumination to conduct speculum exam	-	-	-	*NA*	-	-
Sufficient No of speculums	-	-	+	*NA*	+	+
No stock outs in past 12 months of:						
Male condoms	+	2/3	+	*NA*	+	+
FP methods	+	-	-	*NA*	-	*NA*
Basic STI drugs	+	-	-	*NA*	-	+
All STI drugs	-	-	-	*NA*	-	+
HIV rapid tests	-	2/3	-	*NA*	-	+

HIV/SRH commodities and services available for free at the public health centres included male condoms, hormonal contraceptives, IUD, syndromic STI care and HTS. Female condoms were distributed, but the supply was irregular with many months of stock-outs. The introduction of the contraceptive implant was foreseen at the larger centres, but at the time of the assessment had not yet started. HIV care, including anti-retroviral therapy (ART) and post-exposure prophylaxis (PEP), was offered at all four larger health centres and the hospital. Cervical cancer screening was offered at 2 of the larger health centres and 2 others were foreseen to start screening in the coming years. At none of the health centres were there programmes or services for the prevention or care of SGBV. TOP is sometimes done at the hospital, but only when the mother’s life is endangered. The health centres are not allowed to perform TOP and have no guidelines on how to attend women presenting with an unwanted pregnancy.

The conditions in the public sector were very basic. Space was limited forcing some health centres to offer services by more than one provider in the same room. The consultation rooms were often badly ventilated and illuminated, electricity not always guaranteed, running water sparsely available and toilet facilities dirty or in bad condition. Many consultation rooms functioned without the necessary medical equipment and stock-outs of essential supplies occurred regularly. The limitations in terms of infrastructure and staffing resulted in long waiting queues and limited consultation time.

When asked, all health centre managers were in favour of piloting new interventions and reorganising their services to make them more FSW-friendly, but 4 pointed out that this might be challenged by the lack of staff and 3 by the lack of space.

The assessed private clinic could offer family planning on request, but it was rarely requested because the cost was prohibitive for most clients. HTS was offered, but mostly in a diagnostic or prevention of mother-to-child context. STI care was offered as part of the curative services and male condoms were sold at the clinic’s pharmacy at a commercial price. HIV care was not offered, because of little demand. Cervical cancer screening, TOP services and SGBV services were not included in the package of services offered.

The private clinic had much better conditions in terms of staff, space, equipment and supplies. Clients were attended only by medical doctors, with nursing staff in a supportive role, in spacious, air-conditioned, well-illuminated, well-equipped consultation rooms. The clinic purchased their drugs and supplies themselves on the private market and had no stock-outs.

The Night Clinic offered family planning (only oral and injectable contraceptives), male condoms, STI care, HTS and syphilis screening (using a rapid test), all free of charge. At the time of the assessment, the clinic was in the process of being moved from two containers to a newly built clinic. The new clinic had ample space, was air-conditioned, well illuminated and well equipped. The clinic commodities were supplied by the public health sector and suffered from the same stock-outs. The clinic was operated on week days after hours by three nurses who worked during the day time in the public health sector.

#### HIV/SRH Provider Interviews (Table 2)

**Table 2:** Provider interviews

Department	MCH (*N =* 6)	HTS (*N =* 4)	OPD (*N =* 5)	Chronic disease unit (*N =* 5)	Total (*N =* 18)
Thinks FSWs use the clinic	-	3	1	2	6
Says FSWs disclose	-	1	-	1	2
Best form to provide services to FSWs					
At this clinic	-	-	-	1	1
At a FSW-specific clinic	-	-	-	-	
A combination of both	2	3	1	1	7
No opinion	4	1	4	3	10
Routinely asks about sexual practices					
Yes	6	4	4	5	17
Depends on the patient	-	-	1	-	1
Feels comfortable asking about sexual practices					
Uncomfortable	-	-	1	1	1
Comfortable	5	4	4	3	15
Very comfortable	1	-	-	1	2
Feels comfortable dealing with FSWs					
Comfortable	-	3	1	1	5
Very comfortable	1	-	1	1	3
Never dealt with FSWs	5	1	3	3	10
Discusses condoms with:					
Less than half of the clients	1	-	2	1	3
Half of the clients	-	1	3	3	6
More than half of the clients	-	-	-	1	1
Almost every client	4	3	-	-	7
Gave out condoms during the last week	5	3	4	5	15
Doesn’t know of any guidelines for unwanted pregnancies	6	3	5	5	17
Routinely address SGBV	2	1	-	-	3
Actions taken for victims of SGBV					
Counselling	4	1	3	3	10
Provide PEP	3	1	4	4	10
Refer to a support group	3	2	2	-	6
Depends who the perpetrator is	3	-	1	1	5
Inform the health facility manager	-	1	-	-	1

In total 18 providers were interviewed, 17 providing services at the 5 public health centres and 1 at the obstetrics department of the hospital. Five providers provided services at the FP department, 4 at the HTS unit, 3 at the out-patient department (OPD), 3 at the chronic disease department, and 2 both at the OPD and chronic disease department. The majority of the providers were women (12/18), they had a median age of 37.5 years, were 11 years in service and 4.5 years at the current department. Most of them (11) were nurses or medical agents who followed a 3-years course and have a degree equivalent to 12th grade, 4 were higher level nurses or medical officers who followed a 3-years course after completing secondary school, and 3 were lay counsellors.

Most providers (16/18) said that FSWs do not disclose as such and 12/18 said that therefore they do not know the extent to which FSWs use the health facility. When asked what would be the best approach to provide services to FSWs, either at the health facility, through targeted services, or a combination of both, most providers (10/18) had no opinion on the matter and those that had, mostly (7/18) were in favour of a combined approach.

The providers were asked about different practices when attending to HIV/SRH clients. Almost all providers (17/18) claimed they routinely ask about sexual behaviour (number of partners, type of sexual intercourse). Most also feel comfortable doing this, and all those who were aware that they ever dealt with a FSW were comfortable dealing with a FSW. Most providers (14/18) stated that they discuss condoms with at least half of their clients and almost all (17/18) that they gave out condoms in the past week.

Almost all providers (17/18) confirmed that there were no instructions what to do when a woman presents with an unwanted pregnancy and the actions taken greatly varied from provider to provider. Referring for TOP was not mentioned by any of the providers.

The providers admitted that they do not routinely address SGBV during a HIV/SRH consultation, and only inquire about this when there are obvious signs of violence. The actions taken when a woman is discovered to be victim of SGBV vary among providers, indicating the lack of a standardized approach.

#### HIV/SRH client exit interviews (Table 3)

**Table 3:** HIV/SRH client exit interviews

SRH service	FP (*N =* 37)	HTS (*N =* 26)	HIV care (*N =* 25)	STI care (*N =* 9)	CCS (*N =* 2)	Total (*N =* 99)
Socio-demographic characteristics and sexual behaviour						
Median age	26	24.5	30	27	41	27
Not married/cohabiting	10.8 %	38.5 %	36.0 %	33.3 %	0.0 %	26.3 %
Has more than one sexual partner	2.7 %	3.9 %	4.0 %	11.1 %	0.0 %	4.0 %
Ever exchanged sex for money or goods	8.1 %	11.5 %	12.0 %	33.3 %	0.0 %	12.1 %
Sex work in the last 6 months	0.0 %	3.9 %	8.0 %	11.1 %	0.0 %	4.0 %
Median amount of time spent at the health facility (minutes)	90	90	150	76.5	90	120
Appreciation of the quality of the received services						
Lack of privacy	13.5 %	12.0 %	14.7 %	11.1 %	0.0 %	13.4 %
What needs to be improved most:						
Nothing/ no answer	40.5 %	42.3 %	20.0 %	66.7 %	0.0 %	37.4 %
Infrastructure	25.0 %	23.1 %	8.0 %	0.0 %	50.0 %	18.2 %
Faster attendance	5.6 %	7.7 %	32.0 %	10.0 %	0.0 %	13.1 %
Better equipment	13.9 %	7.7 %	4.0 %	0.0 %	50.0 %	9.1 %
Better attendance	5.6 %	3.8 %	16.0 %	10.0 %	0.0 %	8.1 %
More staff	2.8 %	3.8 %	16.0 %	10.0 %	0.0 %	7.1 %
Other	2.7 %	0.0 %	4.0 %	0.0 %	0.0 %	2.0 %
Talked about HIV prevention	48.7 %	65.4 %	52.0 %	44.4 %	100.0 %	54.6 %
Topics that were addressed:						
Condoms	46.0 %	61.5 %	48.0 %	44.4 %	100.0 %	51.5 %
Fidelity	27.0 %	38.5 %	32.0 %	22.2 %	100.0 %	32.3 %
Received condoms	21.6 %	15.4 %	4.0 %	0.0 %	0.0 %	13.1 %

A total of 99 clients were interviewed, of which 37 were from FP services, 26 from HTS services, 25 from HIV care services, 9 from STI care services and 2 from cervical cancer screening services. The median age of participants was 27 years and 26 % were not married or living with a steady partner. Four percent reported having more than one sexual partner, 12 % stated that they had ever exchanged sex for money or goods and 4 % that they exchanged sex for money or goods at least 3 times in the last 6 months.

Participants spent on average 2 h at the health facility. When asked if their privacy had been respected during the consultation, a substantial proportion (13 %) said it had not, and when asked what is most important to be improved, the most common answers were the infrastructure (18 %), the attendance time (13 %), the quality of the equipment (9 %), the quality of attendance (8 %) and the number of staff (7 %).

When asked if the provider had talked about STI/HIV prevention, 55 % reported he or she did. The messages most commonly given were about male condoms (94 % of those where STI/HIV prevention was addressed) and fidelity (56 %). During their visit, 13 % had received (male) condoms. Most women who received condoms received these from the FP department (8/13), while very few users of services where condoms are expected to be routinely given for STI/HIV prevention (HIV care and STI care) received condoms (2/34).

## Discussion

The objective of the situation analysis was to document what HIV/SRH services were available in the Tete/Moatize area, and to what extent they were adapted to the needs of high-risk women such as FSWs, to better guide a planned intervention to improve access to HIV/SRH services for FSWs.

In the study area, there were three providers of HIV/SRH services: the public sector, a number of private for-profit clinics and a clinic specifically targeting sex workers and other high-risk populations.

The public health sector was by far the most important provider of HIV/SRH services. It offered most essential HIV/SRH services, although that some important services were not (yet) or insufficiently available, such as the contraceptive implant, female condoms, cervical cancer screening, SGBV services and TOP. However, the offered services were not at all adapted to the specific needs of women with a high-risk behaviour such as FSWs. This despite the fact that a substantial proportion of the HIV/SRH clients had risk factors or reported to have engaged in sex work.

Internationally, FSWs are increasingly being recognised as a key population in the fight against HIV, and several guidelines have in past years been developed on how to implement HIV/SRH programmes with female sex workers [18, 19]. These guidelines are however only slowly impacting national-level policy making, and most sex work programmes in Africa have limited coverage and a narrow scope of services [10, 20]. According a 2014 UNAIDS report, only a third of countries had sex worker risk reduction programmes, varying in quality and reach [9]. In Mozambique, it is clearly under pressure from the international community that the Ministry of Health is gradually giving more attention to key populations. It will be important to encourage this process and further develop strategies and guidelines adapted to the needs of sex workers, and other key populations.

Public health providers insufficiently assessed sexual behaviour and risks, and were not identifying FSWs who attend their services. While providers claimed that they routinely ask about sexual behaviour and give out condoms, only half of the clients reported that the provider had addressed HIV prevention and few had received condoms. Most providers did not know if FSWs attended their services, indicating that they do not pro-actively assess if a client is at high risk. There is clearly a need to improve the providers’ skills in assessing risk behaviour and provide FSW-appropriate services.

The assessed public health facilities faced significant shortages in the conditions under which the services are offered. Mozambique is a resource-limited country, ranking 180/188 in the 2015 human development index [21], and these shortages are a nation-wide problem [22, 23]. This is a reality that will not easily change and that has to be taken into account when deciding how to best ensure access to quality HIV/SRH care for FSWs.

Conditions were much better at the private clinic but it offered only a few services, at relatively high cost. A greater role of the private sector in the provision of SRH services is often encouraged, in particular in countries where this sector is an important health care provider [24, 25]. In Mozambique however, the private health sector is a relatively recent phenomenon and has still limited coverage [23]. In our context, it appears therefore not to be a valid alternative to the public sector.

The only alternative was the Night Clinic, a clinic established as part of a an HIV prevention project along transport corridors targeting FSWs and truck drivers, and jointly operated by an NGO and the government [13]. It had better conditions in terms of infrastructure, equipment and staffing, and provided services adapted to FSWs’ needs, but had a more limited geographical range and scope of services.

There is no international consensus on what is the best approach to guarantee that FSWs have sufficient access to HIV/SRH services. Establishing parallel services specifically for key populations is generally the approach preferred by FSWs themselves and that best ensures access and appropriate care [11–13, 26], but it is more costly, less sustainable (because of often having little government support and relying on project-based funding), and has a risk of stigmatisation [10–12, 27, 28]. In Mozambique, there was no government policy on how to best ensure access to health services for marginalised populations such as FSWs. Government policy makers clearly favoured an integrated approach where access to the public health services is ensured by making them more FSW-friendly. Projects financed by the international community have established over the past years several FSW-specific health services in the country, mostly in the form of small stand-alone clinics, such as the Night Clinic [13, 29]. Government policy makers accepted the existence of these services, but mostly as a temporary measure until access to the public health system is improved. The HIV/SRH care providers had no outspoken opinion on the subject, and mostly favoured an approach where FSWs are attended both at the public services and at separate clinics. Health centre managers were all in favour of piloting new interventions to make their services more FSW-friendly, but warned that this might be hampered by their lack of staff and space.

Each of the components of our study has certain limitations, such as reporting bias when conducting face-to-face interviews. However, we believe that we reduced these limitations to a minimum by using a mixed-methods design, and reaching integrated conclusions by comparing the results of complimentary methods. Together with the findings of the other part of the baseline analysis, that assesses the needs from the FSWs’ perspective, it will provide guidance for the development of an appropriate intervention package.

## Conclusion

The public health sector is the most important provider of HIV/SRH services in the area, with the private sector playing only a marginal role and a project-funded drop-in clinic targeting key populations offering a smaller range of services to the FSW population in only part of the area. The public HIV/SRH services are not adapted to the needs of female sex workers and face serious limitations in terms of staff, space, equipment and supplies. There is a need to improve the availability of quality HIV/SRH services and to develop guidelines for care adapted to the specific context of FSWs. This can be done by either expanding the range of services and the coverage of the stand-alone clinic and/or by improving access to adapted care at the public health services and ensure a minimum quality of care. The government of Mozambique needs to develop a strategy that guarantees access to services for FSWs that are both effective and sustainable.

## Abbreviations

ART, antiretroviral therapy; DIFFER, diagonal interventions to fast-forward expanded reproductive health; FP, family planning; FSW, female sex worker; HIV, human immunodeficiency virus; HTS, HIV testing services; IUD, intra-uterine device; KI, key informant; MCH, maternal and child health; NGO, non-governmental organisation; OPD, out-patient department; PEP, post exposure prophylaxis; SGBV, sexual and gender-based violence; SRH, sexual and reproductive health; STI, sexually transmitted infections; TOP, termination of pregnancy

## Additional file

Additional file 1: Table S1. Summary of policy analysis findings. (DOCX 14 kb)

## References

[1] Baral S, Beyrer C, Muessig K, Poteat T, Wirtz AL, Decker MR (2012). Burden of HIV among female sex workers in low-income and middle-income countries: a systematic review and meta-analysis. Lancet Infectious Diseases.

[2] Pruss-Ustun A, Wolf J, Driscoll T, Degenhardt L, Neira M, Calleja JMG. HIV Due to Female Sex Work: Regional and Global Estimates. Plos One. 2013;8(5). 10.1371/journal.pone.0063476 PMC3662690 23717432

[3] Scorgie F, Chersich MF, Ntaganira I, Gerbase A, Lule F, Lo YR (2012). Socio-Demographic Characteristics and Behavioral Risk Factors of Female Sex Workers in Sub-Saharan Africa: A Systematic Review. Aids and Behavior.

[4] Morineau G, Neilsen G, Heng S, Phimpachan C, Mustikawati DE (2011). Falling through the cracks: contraceptive needs of female sex workers in Cambodia and Laos. Contraception.

[5] Schwartz S, Papworth E, Thiam-Niangoin M, Abo K, Drame F, Diouf D (2015). An Urgent Need for Integration of Family Planning Services Into HIV Care: The High Burden of Unplanned Pregnancy, Termination of Pregnancy, and Limited Contraception Use Among Female Sex Workers in Cote d’Ivoire. Jaids-Journal of Acquired Immune Deficiency Syndromes.

[6] Sutherland EG, Alaii J, Tsui S, Luchters S, Okal J, King’ola N (2011). Contraceptive needs of female sex workers in Kenya -- A cross-sectional study. Eur. J. Contracept. Reprod. Health Care.

[7] Decker MR, Crago AL, Chu SKH, Sherman SG, Seshu MS, Buthelezi K (2015). Human rights violations against sex workers: burden and effect on HIV. Lancet.

[8] Scorgie F, Vasey K, Harper E, Richter M, Nare P, Maseko S, et al. Human rights abuses and collective resilience among sex workers in four African countries: a qualitative study. Globalization and Health. 2013;9 10.1186/1744-8603-9-33 PMC3750273 23889941

[9] UNAIDS (2014). The Gap Report.

[10] Dhana A, Luchters S, Moore L, Lafort Y, Roy A, Scorgie F, et al. Systematic review of facility-based sexual and reproductive health services for female sex workers in Africa. Globalization and Health. 2014;10 10.1186/1744-8603-10-46 PMC4070634 24916010

[11] Coulibaly A, Keita BD, Henry E, Trenado E (2014). Facilitating access to care for most-at-risk populations: The Bamako night sexual health clinic experience (Mali). Sante Publique.

[12] Stadler J, Delany S (2006). The ‘healthy brothel’: The context of clinical services for sex workers in Hillbrow, South Africa. Cult Health Sex.

[13] Lafort Y, Geelhoed D, Cumba L, Lazaro CDM, Delva W, Luchters S, et al. Reproductive health services for populations at high risk of HIV: Performance of a night clinic in Tete province, Mozambique. BMC Health Services Research. 2010;10 10.1186/1472-6963-10-144 PMC2890643 20507644

[14] ICRH. The DIFFER Project. Available from: http://differproject.eu/. Accessed 12 May 2016.

[15] Creswell JW, Plano Clark VL (2011). Designing and Conducting Mixed Methods Research.

[16] Curry LA, Krumholz HM, O’Cathain A, Clark VLP, Cherlin E, Bradley EH (2013). Mixed Methods in Biomedical and Health Services Research. Circ. Cardiovasc. Qual. Outcomes.

[17] Patton MQ (2002). Qualitative research and evaluation methods.

[18] World Health Organization, UNPF, Joint United Nations Programme on HIV/AIDS, Global Network of Sex Work Projects, The World Bank (2013). Implementing comprehensive HIV/STI programmes with sex workers: practical approaches from collaborative interventions.

[19] World Health Organization (2014). Consolidated guidelines on HIV prevention, diagnosis, treatment and care for key populations.

[20] Alary M, Lowndes CM, Van de Perre P, Behanzin L, Batona G, Guedou FA (2013). Scale-up of combination prevention and antiretroviral therapy for female sex workers in West Africa: time for action. Aids.

[21] UNDP (2015). Human Development Report 2015.

[22] IHP+ (2013). Joint Assessment of the Mozambican Health Sector Strategic Plan (PESS, 2014).

[23] MISAU. Relatório da Revisão do Sector de Saúde. Maputo, Mozambique: Mozambican Ministry of Health, República de MoçambiqueMinistério de Saúde; 2012.

[24] Madhavan S, Bishai D (2010). Private Sector Engagement in Sexual and Reproductive Health and Maternal and Neonatal Health - A Review of the Evidence. Johns Hopkins Bloomberg School of Public Health, Department of Population F, and Reproductive Health.

[25] Peters DH, Mirchandani GG, Hansen PM (2004). Strategies for engaging the private sector in sexual and reproductive health: how effective are they?. Health Policy Plan.

[26] Scorgie F, Nakato D, Harper E, Richter M, Maseko S, Nare P (2013). ‘We are despised in the hospitals’: sex workers’ experiences of accessing health care in four African countries. Cult Health Sex.

[27] Beyrer C, Baral S, Kerrigan D, El-Bassel N, Bekker LG, Celentano DD (2011). Expanding the Space: Inclusion of Most-at-Risk Populations in HIV Prevention, Treatment, and Care Services. Jaids-Journal of Acquired Immune Deficiency Syndromes.

[28] Vuylsteke B, Traore M, Mah-Bi G, Konan Y, Ghys P, Diarra J (2004). Quality of sexually transmitted infections services for female sex workers in Abidjan, Cote d’Ivoire. Trop Med Int Health.

[29] FHI-360. ROADS to a Healthy Future (ROADS II) in Mozambique. Available from: http://www.fhi360.org/projects/roads-healthy-future-roads-ii-mozambique. Accessed 12 May 2016.

