# Supplementary material for: Are HIV and reproductive health services adapted to the needs of female sex workers? Results of a policy and situational analysis in Tete, Mozambique
Source: BMC Health Serv Res. 2016 Jul 26;16:301. doi: 10.1186/s12913-016-1551-y (PMC4960856; doi:10.1186/s12913-016-1551-y)
Supplement: Additional file 1: Table S1. — Summary of policy analysis findings. (DOCX 14 kb) [file 12913_2016_1551_MOESM1_ESM.docx]

Table 1: Summary of policy analysis findings

| Service | Strategic documents | Summary of information extracted from the strategic documents and provided by the Key Informants |
| --- | --- | --- |
| Condom distribution | No strategic documents | Male condoms are distributed for free by the public health sector at the Out-Patient and the Maternal and Child Health Department of all health facilities, mostly in the context of STI care and Family Planning. Female condoms are being purchased by the Ministry of Health for about two years, but their distribution is still very irregular. |
| Family Planning | Five-year strategy (2011-2015)  Technical guidelines (2011) | Family planning is offered for free at all public health facilities. The pill, injectable contraceptives and male condoms are offered at all health facilities. IUD is only offered where there is a nurse who knows how to insert them. Tubal ligation and male sterilisation is only offered at hospitals with surgical facilities. Implant is recently offered, but only at some health facilities in the major urban areas. The policy of the MoH is to promote long-acting FP methods, such as IUD and implant. |
| STI care | Technical guidelines (2006) | STI care is offered integrated at the OPD, but also at all services of the MCH department. Syndromic management is recommended only in health facilities without laboratory facilities. The decision to treat women with vaginal discharge for gonorrhoea and chlamydia is contingent on clinical criteria being met. A speculum exam is recommended and treatment to be given only in the presence of either cervical signs or smelly/yellow discharge. If speculum exam is not possible, all women with a clinically suspicious vaginal discharge should be treated. |
| Cervical cancer screening | Technical guidelines | Cervical cancer screening is currently only offered at a limited number of health facilities. The programme is gradually being expanded. The principle is to have a chain of a primary health care facility, a secondary health facility and a specialised health facility. The primary facility does mobilisation and a first screening, with VIA. If positive, localised lesions are treated at the secondary facility and advanced lesions at a hospital. When deciding where to initiate screening, this chain is taken into consideration. The guidelines mention that ‘all women between 30 and 55’ need to be screened. |
| HIV testing & counselling | No strategic documents | There are no guidelines, but the HTC training manual explains the national testing algorithm. A combination of 2 rapid tests is used: a first screening with Determine and confirmation of positive results with Unigold. |
| HIV care | Technical guidelines (2009/2010)  Power point presentation of planned revised guidelines | The technical guidelines for HIV treatment are outdated and in the process of being revised. There is no strategic document, but there is a National Acceleration Plan for the Prevention, Diagnosis and Treatment of HIV being developed. There are also guidelines being developed on psycho-social support and positive prevention among PLHIV. There exists a document on positive prevention that was developed in 2010. HIV care is decentralised up to the health centre level, where it is offered at ‘chronic care units’ and it is foreseen to further decentralise it to the MCH departments. |
| SGBV care | Power point presentation of planned guidelines | SGBV services in the country are still mostly offered in the context of specific projects and the situation varies therefore substantially across the country. In theory each provincial hospital should have an SGBV unit. Most SGBV initiatives are in the form of a network, involving the police department, the ministry of health and the ministry of women and social affairs. There exists a strategy developed by the MoH, that describes the care flow, clinical conduct, links with the community, links with the police …. The document is currently in the process of being officially approved. |
| Termination of pregnancy | No strategic documents | Services for unwanted pregnancies exist in some hospitals, but they are not formally authorised. It is not clear what the indications are for termination of pregnancy (TOP), but in practice it is rarely done and if it is done, mostly because the life of the mother is endangered. Unsafe abortions happen frequently, even at hospitals that are allowed to perform TOP. From a legal perspective, TOP is currently illegal in all situations. The penal code is currently being revised and there is a serious debate about abortion. |
| HIV/SRH services for key populations | Draft of orienting document | None of the SRH policies, strategies or guidelines specifically mention key populations. Recently, under pressure from the international community, there is larger attention on key populations, a working group was created and guidelines are being developed for attending key populations at health facilities. In the draft new National Acceleration Plan for the Prevention, Diagnosis and Treatment of HIV, key populations are to be targeted for HTC and one of the issues being discussed is treatment as prevention in key populations. |
